# The *S. pombe* Translation Initiation Factor eIF4G Is Sumoylated and Associates with the SUMO Protease Ulp2

**DOI:** 10.1371/journal.pone.0094182

**Published:** 2014-05-12

**Authors:** Jirapas Jongjitwimol, Min Feng, Lihong Zhou, Oliver Wilkinson, Lauren Small, Robert Baldock, Deborah L. Taylor, Duncan Smith, Lucas D. Bowler, Simon J. Morley, Felicity Z. Watts

**Affiliations:** 1 Genome Damage and Stability Centre, School of Life Sciences, University of Sussex, Falmer, Brighton, United Kingdom; 2 Paterson Institute for Cancer Research, The University of Manchester, Manchester, United Kingdom; 3 Biochemistry and Biomedical Sciences, School of Life Sciences, University of Sussex, Falmer, Brighton, United Kingdom; The John Curtin School of Medical Research, Australia

## Abstract

SUMO is a small post-translational modifier, that is attached to lysine residues in target proteins. It acts by altering protein-protein interactions, protein localisation and protein activity. SUMO chains can also act as substrates for ubiquitination, resulting in proteasome-mediated degradation of the target protein. SUMO is removed from target proteins by one of a number of specific proteases. The processes of sumoylation and desumoylation have well documented roles in DNA metabolism and in the maintenance of chromatin structure. To further analyse the role of this modification, we have purified protein complexes containing the *S. pombe* SUMO protease, Ulp2. These complexes contain proteins required for ribosome biogenesis, RNA stability and protein synthesis. Here we have focussed on two translation initiation factors that we identified as co-purifying with Ulp2, eIF4G and eIF3h. We demonstrate that eIF4G, but not eIF3h, is sumoylated. This modification is increased under conditions that produce cytoplasmic stress granules. Consistent with this we observe partial co-localisation of eIF4G and SUMO in stressed cells. Using HeLa cells, we demonstrate that human eIF4GI is also sumoylated; *in vitro* studies indicate that human eIF4GI is modified on K1368 and K1588, that are located in the C-terminal eIF4A- and Mnk-binding sites respectively.

## Introduction

Sumoylation is a post-translational protein modification that is required for numerous processes within cells, including transcription, chromosome segregation, DNA damage responses, cell signalling and meiosis (reviewed in [1]–[7]). At the molecular level it functions by altering the surface of target molecules to affect protein-protein interactions e.g. of PCNA (proliferating cell nuclear antigen) and Srs2 (a DNA helicase) [8], [9], by altering the intracellular localisation of proteins e.g. of RanGAP [10], or by changing the conformation of target proteins (e.g. in the case of thymine DNA glycosylase [11]). SUMO chains attached to target proteins can also be ubiquitinated and thus result in proteolysis of the target.

SUMO is a small ubiquitin-like modifier that is attached to lysine residues in target proteins. The yeasts *Schizosaccharomyces pombe* and *Saccharomyces cerevisiae* both have a single gene for SUMO: *pmt3* and *SMT3*, respectively, while mammals have four, SUMO-1, -2, -3 and -4 (although the role of SUMO-4 is not well defined). SUMO-2 and -3 are 97% identical to each other and about 50% identical to SUMO-1 (reviewed in [1]). SUMO is produced as a precursor protein that needs to be cleaved into the mature form in order to act as a substrate in the sumoylation reaction. Processing of SUMO requires a specific SUMO-protease [12]–[14], and involves the removal of a small number of amino acids from the C-terminus of precursor SUMO to reveal a Gly-Gly motif. Mature SUMO is then activated by the formation of a thioester bond between the C-terminal glycine residue and a cysteine residue in one subunit of the SUMO activating enzyme (E1). From here SUMO is passed to the SUMO conjugating enzyme (E2), where it again forms a thioester bond with another cysteine residue. SUMO can then be attached to one or more lysine residues in the target protein. In some cases, one of a small number of SUMO ligases (E3) is required for conjugation. In many cases the lysine is contained within the consensus motif ψKxE, where ψ is a hydrophobic amino acid, and x is any amino acid. SUMO can be added to target proteins as a monomer or as poly-SUMO in the form of chains. The removal of SUMO from target proteins or dismantling of SUMO chains occurs via the action of SUMO-specific proteases [14], [15].

In *S. cerevisiae* there are two SUMO proteases, Ulp1 and Ulp2, both of which can deconjugate SUMO from target proteins, but which have different target specificities [12]. Only Ulp1 is capable of processing precursor SUMO to the mature form [12], [15]. Ulp1 and Ulp2 are differently localised within the cell: Ulp1 is located at nuclear pores, while Ulp2 is located mainly within the nucleus [15]. Mammalian cells have six SUMO-specific proteases (SENPs). These are also differentially localised within cells and have different abilities to cleave precursor SUMO and to deconjugate SUMO from targets e.g. [16], [17]. The *S. pombe* Ulp1 protease has been characterised and shown to process SUMO to the mature form, and like *S. cerevisiae* Ulp1, to be located at the nuclear periphery [13]. However, little is known about Ulp2 in this organism.

Translation initiation factors, which play key roles in cell survival and oncogenesis [18]–[22], can be modified by sumoylation [6], [7], [23]–[31]. Protein synthesis is carried out in three stages (initiation, elongation and termination), with the initiation stage of translation generally accepted as a major site of regulation of gene expression in mammalian cells [18]–[22]. This step in protein synthesis is regulated by a family of proteins, the initiation factors [18], [21], [22] which interact with each other and the mRNA. These proteins modulate the binding of mRNA to the ribosome, a process facilitated by the assembly of the cap binding protein (eIF4E), a helicase (eIF4A) and a scaffold protein (eIF4G), to form the eIF4F complex (eIF4E/eIF4A/eIF4G). The eIF4G scaffold protein possesses domains that interact with eIF4E, eIF4A, eIF3 and the poly(A) binding protein (PABP) [18], [20]–[22]. The activity of the eIF4F complex is regulated by a family of proteins, the eIF4E binding proteins (4E-BPs). Using a conserved motif, 4E-BPs compete with eIF4G for a common surface on eIF4E and inhibit eIF4F assembly. In mammalian cells, activation of the mechanistic target of rapamycin (mTORC1) leads to the multi-site phosphorylation of 4E-BP1 [18], [22], [32] preventing 4E-BP1 from binding to eIF4E and thereby allowing formation of the eIF4F initiation complex and ribosomal recruitment of mRNA [18], [21], [22]. More recently, phosphorylated human eIF4E has been shown to be modified by sumoylation on five lysine residues [33]. Consistent with a role in modulating protein-protein interactions [34], sumoylation did not interfere with mRNA recognition but enhanced eIF4F complex level assembly on the mRNA cap, promoting the expression of ornithine decarboxylase, c-myc and Bcl-2, thereby driving the anti-apoptotic and oncogenic activity of eIF4E [33].

Since the majority of SUMO in cells is present in the nucleus, much of the work undertaken to understand the role of sumoylation has focussed on its role in regulating events associated with DNA metabolism, such as the maintenance of chromatin structure, recombination and DNA damage responses [3], [5], [8], [9]. More recently it has been demonstrated that sumoylation is required in the nucleolus to regulate ribosome biogenesis e.g. [35]. In order to obtain a fuller understanding of the role of sumoylation we have begun to investigate the protein-protein interactions and localisation of the mostly uncharacterised *S. pombe* SUMO protease, Ulp2. Our results from gel filtration and immunofluorescence studies indicate that Ulp2 is present in at least two high Mr complexes, which are distinct from the nuclear pore complex that contains Ulp1. We demonstrate that it co-purifies with a number of proteins, many of which are involved in RNA metabolism or protein synthesis. We have investigated whether two of these proteins, eIF4G and eIF3h, are sumoylated, with the result that we observe SUMO modification of eIF4G but not eIF3h. Exposure of cells to conditions that lead to the formation of stress granules, results in increased sumoylation of eIF4G, and partial co-localisation of eIF4G and SUMO in the cytoplasm. Finally, we demonstrate that human eIF4G is sumoylated in HeLa cells, by both SUMO-1 and SUMO-2.

## Materials and Methods

### Strains and plasmids

The strains used in this work are described in Table 1. The strains containing myc-, HA or TAP-tagged *ulp1*, *ulp2*, *pli1*, *eIF4G* and *eIF3h* were created using the method of Bahler et al [36]. pREP41-His-SUMO was constructed by cloning the *pmt3* ORF into pREP41-His (created in this study). The *S. pombe* and human eIF4G and eIF4GI constructs, Sp C-term, N-FAG, M-FAG and C-FAG contain different fragments of the eIF4G/eIF4GI Orfs cloned into pET15b [37]. HeLa cell lines stably transfected with His-SUMO-1 and His-SUMO-2 were gifts from Prof R Hay (University of Dundee) [38], [39].

**Table 1:** List of strains.

Strain	Genotype	Reference
Sp.011	*ade6-704*, *leu1-32*, *ura4-D18*, *h^−^*	[72]
Sp.611	*ulp1-myc:kan*, *ade6-704*, *leu1-32*, *ura4-D18*, *h^−^*	This study
Sp.614	*ulp2-myc:kan*, *ade6-704*, *leu1-32*, *ura4-D18*, *h^−^*	This study
Sp.658	*ulp1::ura4, ade6-704*, *leu1-32*, *ura4-D18*, *h^−^*	[13]
Sp.723	*pli1-myc:kan*, *ade6-704*, *leu1-32*, *ura4-D18*, *h^−^*	This study
Sp.874	*pmt3-GG:ura4, ade6-704*, *leu1-32*, *ura4-D18*, *h^−^*	This study
sp.851	*ulp1::ura4, pmt3-GG:ura4*, *ade6-704*, *leu1-32*, *ura4-D18, h^−^*	This study
sp.855	*ulp2::ura4, pmt3-GG:ura4*, *ade6-704*, *leu1-32*, *ura4-D18, h^−^*	This study
Sp.1470	*ulp2-TAP, ade6-704*, *leu1-32*, *ura4-D18*, *h^−^*	This study
Sp.2047	*eIF3h-HA:Nat*, *ade6-704*, *leu1-32*, *ura4-D18*, *h^+^*	This study
Sp.2048	*ulp2-myc*:*kan*, *eIF3h-HA:Nat*, *ade6-704*, *leu1-32*, *ura4-D18*, *h^−^*	This study
Sp.2068	*ulp2-myc:kan*, *eIF4G-HA:Nat*, *ade6-704*, *leu1-32*, *ura4-D18*, *h^−^*	This study
Sp.2085	*ulp2::kan*, *ade6-704*, *leu1-32*, *ura4-D18*, *h^−^*	This study
Sp.2088	*eIF4G-HA:Nat*, *ade6-704*, *leu1-32*, *ura4-D18*, *h^+^*	This study

### Ulp2 expression and assay

The *ulp2* ORF was amplified from cDNA, by PCR and cloned into pFastBacHTa (GibcoBRL). Recombinant baculoviruses were generated according to GibcoBRL instructions. 50 ml infected cells were lysed in 50 mM Tris HCl pH 8, 5 mM β-mercaptoethanol, 1% nonidet, 1 mM PMSF. Ulp2 protein was purified using Talon resin. Ulp2 activity assays were conducted as described for Ulp1 [13].

### Protein purification methods

His-tagged SUMO was recovered from *S. pombe* and human whole cell extracts under denaturing conditions with Ni^2+^ agarose beads. Cell extracts were prepared as follows: 10^8^ cells (*S. pombe*) or 6–8×10^6^ cells (Hela) were washed in ice cold water before being lysed by vortexing in 1.85 M NaOH, 7.5% v/v β-mercaptoethanol. The lysate was incubated on ice for 20 min after which TCA was added to a final concentration of 25%. Following a further 20 min incubation on ice, precipitated proteins were collected by centrifugation and resuspended and solubilised in 1 ml buffer A (6 M guanidinium HCl, 0.1 M NaH_2_PO_4_, 10 mM Tris-HCl, pH 8). Insoluble material was removed by centrifugation. The cell extract was then incubated with Ni^2+^ agarose (Novagen) in Buffer A in the presence of 0.05% Tween-20, 150 mM imidazole. Purification on Ni^2+^ agarose was carried out according to the manufacturer's instructions. Samples were analysed by SDS-PAGE and Western blotting. His-tagged *S. pombe* and human eIF4GI fragments for *in vitro* sumoylation assays were purified from *E. coli* using Ni^2+^ agarose according to the manufacturer's instructions.

For gel filtration, 200 ml logarithmically growing cells were harvested, washed and then broken in 1 ml ice cold lysis buffer (45 mM HEPES pH 7.8, 300 mM KCl, 5 mM MgCl_2_, 5 mM EDTA, 5 mM EGTA, 12 mM NaF, 10% glycerol, 80 mM β-glycerophosphate, 0.1 mM sodium orthovanadate, 1 mM PMSF, 1 mM DTT, supplemented with Roche complete protease inhibitor). The extract was clarified by two rounds of centrifugation at 20,000 rpm for 10 min. 1.5 mg protein was loaded onto either a Superdex 200 or Superose 6 column pre-equilibrated in lysis buffer. 0.5 ml fractions were collected and 15 µl of each was analysed by SDS PAGE.

For TAP-purification, 60 l *ulp2-TAP* cells were grown to mid-log phase, harvested and frozen at −80°C until required. Ulp2-TAP was purified using a modification of the method described by Seraphin et al. [40]. Specifically, the cells were broken in a 6850 freezer mill in 20 mM Tris-HCl, pH 7.5, 10 mM NaCl, 1 mM DTT, 0.5 mM EDTA, 2 mM MgCl_2_, 0.1 mM NaF, 0.1% Nonidet NP40, 1 mM PMSF, 5 mM sodium orthovanadate, 80 mM β-glycerophosphate, 10 mM N-ethylmaleimide, supplemented with Roche complete protease inhibitor. All subsequent procedures were carried out at 4°C. The cell extract was centrifuged twice for 1 h at 10,000 rpm. Samples were pre-cleared by incubation with 200 µl Dynabeads for 30 min to remove proteins that bound non-specifically to the beads. The extracts were incubated with 300 µl IgG-coated Dynabeads for 2 h. The beads were collected and washed extensively before being resuspended in TEV buffer (50 mM Tris-HCl pH 8.0, 0.5 mM EDTA, 1 mM DTT) with 250 units AcTEV protease (Invitrogen) for 3 h. The IgG-coated Dynabeads were removed from the preparation and Ulp2-TAP containing complexes were snap frozen in liquid nitrogen.

### *In vitro* sumoylation assay

Recombinant His-tagged *S. pombe* eIF4G and human eIF4GI fragments were purified from *E. coli* and tested for sumoylation in an *in vitro* sumoylation assay as described elsewhere [41]. SUMO-TRGG (Pmt3-L109R,GG: the mature form of *S. pombe* SUMO containing a trypsin cleavage site immediately upstream of the diglycine motif) was used in the assay to facilitate the identification of the sumoylation sites by mass spectrometry.

### Immunological methods

Western analysis was carried out as described previously [13]. Production of anti-SUMO and anti-eIF4GI (against the KRERK epitope) antisera has been described elsewhere [41], [42], anti-myc antibodies for immunofluorescence were purified from cell supernatant (cell line CRL1729, from ATCC) using protein G-sepharose or were from Santa Cruz (sc-40), anti-HA antisera were from Santa Cruz (sc-7392) and monoclonal anti-tubulin antibodies were from Sigma (T5168). Immunofluorescence was undertaken as described in Moreno et al. [43]. Cells were observed using an Applied Precision Deltavision Spectris microscope using deconvolution software.

### Mass spectrometry

Complexes purified by purification of TAP-Ulp2 were analysed by SDS PAGE. Protein bands were visualised by staining with colloidal Coommassie, excised and subjected to trypsin in-gel digestion essentially as described by Schevchenko et al. [44]. The supernatant from the digested samples was removed and acidified to 0.1% TFA, dried down, and reconstituted in 0.1% TFA prior to LC MS/MS analysis. Each sample was loaded and desalted at a flow rate of 5 µl/min on a C18 trap column (200 µm ID x 1 cm, 5 µm PepMap 100, Dionex) in buffer A (acetonitrile (2% v/v): water (97.9% v/v): formic acid (0.1% v/v)). The tryptic peptides were fractionated on a C18 reverse phase column (75 µm ID x 25 cm, 3 µm PepMap 100, Dionex) using an Ultimate U3000 nano-LC system (Dionex) and a 2 hr linear gradient from 95% buffer A to 50% buffer B (acetonitrile (95% v/v): water (4.9% v/v): formic acid (0.1% v/v) at a flow rate of 300 nl/min. Eluted peptides were directly analysed by tandem mass spectrometry using a LTQ Orbitrap XL hybrid FTMS (ThermoScientific) operated in parallel acquisition IDA mode with nominal resolution of 60,000 (FWHM) at m/z 400 for MS1 and the top six most abundant multiply charged ions being selected for CID fragmentation in the linear ion trap followed by dynamic exclusion for 90 secs.

Derived MS/MS data were searched against the *S. pombe* subset of the UniProt Knowledgebase release 15.13 database using Sequest version SRF v. 5 as implemented in Bioworks v 3.3.1 (Thermo Fisher Scientific), assuming carboxyamidomethylation (Cys), deamidation (Asn and Gln) and oxidation (Met) as variable modifications and using a peptide tolerance of 10 ppm and a fragment ion tolerance of 0.8 Da. One missed cleavage was allowed and filtering criteria used for positive protein identifications were Xcorr values greater than 1.9 for +1 spectra, 2.2 for +2 spectra and 3.75 for +3 spectra and a delta correlation (DCn) cut-off of 0.1.

For the identification of sumoylation sites, reduction and alkylation were instead performed using TCEP and MMTS respectively as previously described [45] and bioinformatics analysis following conversion of LTQ-Orbitrap (.raw) raw data files to Mascot generic format (MGF) via Mascot Distiller (Matrix Science) performed essentially as described by Chicooree et al. [46] using the MASCOT search engine with the UniProt Knowledgebase release 15.13 database with the *S. pombe* subset as selected taxonomy. Precursor ion tolerances were again set at 10 ppm and MS/MS peptide ion tolerance to 0.8 Da, and the same variable modifications assumed. However, two missed trypsin cleavages were allowed.

Following trypsin digestion, cleavage of the SUMO moiety was expected to leave a Gly-Gly isotag on modified residues. The GG isotag (on lys) was accordingly also searched as a variable modification. Following MASCOT searches, putative sites of SUMOylation were noted and the relevant raw MS/MS spectra subsequently examined manually to confirm presence of the modification (the GG isotag).

## Results

### Biochemical characterisation of *S. pombe* Ulp2

**Figure 1:**
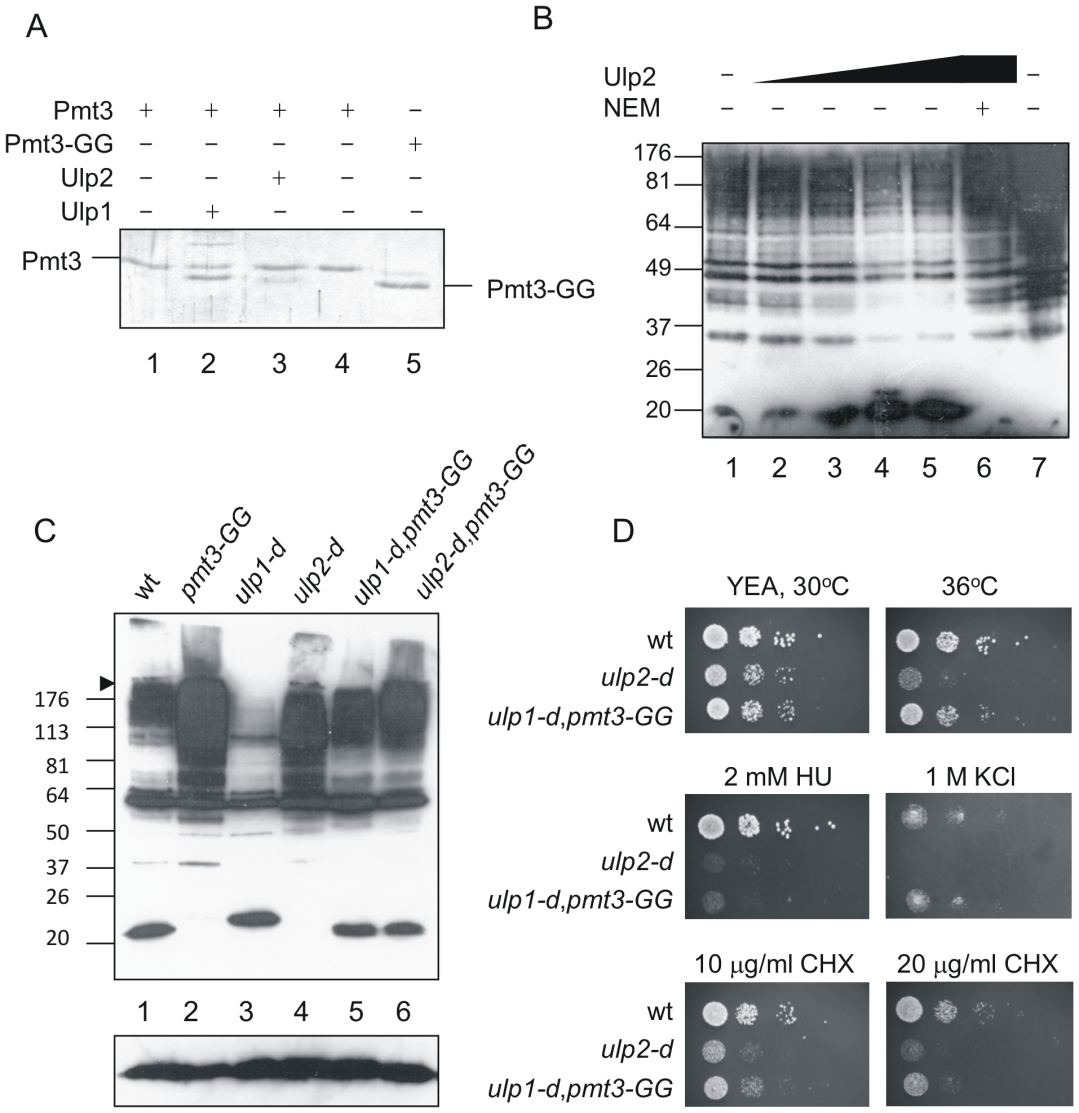
Analysis of Ulp2 function. A. Assay for SUMO-processing activity. Lanes 1–4 contain full length SUMO, lane 5 SUMO-GG. Lanes 1,5, unincubated controls, lanes 2–4 were incubated at 20°C for 2 h following addition of 0.72 µg Ulp1 (lane 2), 2.32 µg Ulp2 (lane 3) or 2 µl buffer (lane 4). Proteins were analysed by SDS PAGE followed by staining with Coommassie Brilliant Blue. B. Assay for de-conjugating activity. *S. pombe* cell extracts were prepared using standard native extraction procedures. Extracts were incubated at 20°C for 2 h (lanes 1–6), lane 1 5 µl of fraction from extract from *E. coli* cells transformed with empty vector, equivalent in volume to the Ulp2-containing fraction from *ulp2*-transformed cells, lane 2 0.6 µg Ulp2, lane 3 1.2 µg Ulp2, lane 4 2.4 µg (5 µl) Ulp2, lane 5 4.8 µg Ulp2, lane 6 1.2 µg Ulp2 pre-incubated with 5 mM NEM, lane 7 total cell extract without incubation at 20°C. Assays were analysed by Western blotting with anti-SUMO antisera. C. Western analysis of total cell extracts using anti-SUMO antisera. Both the separating and stacking gels (6% polyacrylamide in the stacking gel) were blotted. D. Ten microlitre of 10 fold serial dilutions of cells were plated onto YEP agar plates with or without additives as indicated. 10x amount of cells of *ulp2-d* and *ulp1-d,pmt3-GG* were used compared to wild type.

A comparison of the *S. pombe* Ulp2 sequence was made with those of the two *S. cerevisiae* SUMO proteases, Ulp1 and Ulp2 [47]. Since *S. pombe* Ulp2 more closely resembles *S. cerevisiae* Ulp2 (required solely for deconjugating SUMO from high Mr SUMO-containing species) than it does Ulp1 (which is required for both processing and deconjugating), it is likely that the main activity of *S. pombe* Ulp2 is in deconjugating SUMO from sumoylated targets rather than in processing SUMO to the mature form. Before proceeding to analyse the localisation or protein-protein interactions of Ulp2, we first confirmed its proposed biochemical activity. His-tagged Ulp2 was purified from insect cells as described in Materials and Methods. Using assays we described previously [13], we demonstrate that Ulp2 is significantly less able than Ulp1 to process SUMO to the mature form (Figure 1A, lane 2 (Ulp1) and lane 3 (Ulp2)), but is capable of deconjugating SUMO from high Mr species in an N-ethylmaleimide- (NEM)-dependent manner (Figure 1B). These results confirm that like *S. cerevisiae* Ulp2, *S. pombe* Ulp2 is a cysteine protease whose main function is in deconjugating SUMO from target proteins.

### Deletion of the *ulp2* gene results in a severe growth defect and sensitivity to a range of stresses

Deletion of *pmt3* (which encodes SUMO), *hus5* (the gene encoding the SUMO-conjugating enzyme, E2), *rad31* (which encodes one sub-unit of the SUMO activating enzyme, E1) or *ulp1* (another SUMO-specific protease gene) results in severe growth and morphological abnormalities [13], [48]–[50]. We therefore wished to determine whether disrupting the *ulp2* gene has any effect on cell growth or viability. Disruption of the gene is not lethal. However, *ulp2-d* cells form very small colonies and show distinct morphological abnormalities resembling *hus5* and *rad31* mutants (data not shown). Comparison of SUMO-containing species in *ulp1-d* and *ulp2-d* cells (Figure 1C, lanes 3 and 4) supports the notion that the main function of Ulp2 is in the removal or dismantling of high Mr SUMO-containing species, rather than in processing precursor SUMO. Provision of the mature form of SUMO (Pmt3-GG) in *ulp1-d* cells (lane 5) results in the incorporation of SUMO into high Mr species (unlike the situation in *ulp1-d* cells, lane 3), while in *ulp2-d* cells (lane 6), the level of high Mr species is slightly increased.

To begin to identify cellular processes involving Ulp2, we tested whether *ulp2-d* cells are sensitive to the DNA synthesis inhibitor, hydroxyurea (HU) and other stresses (Figure 1D), and compared these responses to those of *ulp1-d*,*pmt3-GG* cells (where the mature form of SUMO is provided, so that cells are only defective in the deconjugating activity of Ulp1). Since *ulp2-d* and *ulp1-d,pmt3-GG* cultures contain a high proportion of dead cells, it was necessary to plate more cells for these strains compared to wild type (approximately 10 fold). These data indicate that *ulp2-d* cells are temperature sensitive, unlike the *ulp1-d*,*pmt3-GG* strain, but similar to the *S. cerevisiae ulp2Δ* strain [51], and sensitive to the DNA synthesis inhibitor, hydroxyurea (HU, 2 mM). They are also sensitive to the protein synthesis inhibitor, cycloheximide (CHX, 10 and 20 µg/ml) and KCl (1 M) indicating that Ulp2 likely has roles in numerous cellular processes.

### Ulp2 is present in high molecular weight complexes

**Figure 2:**
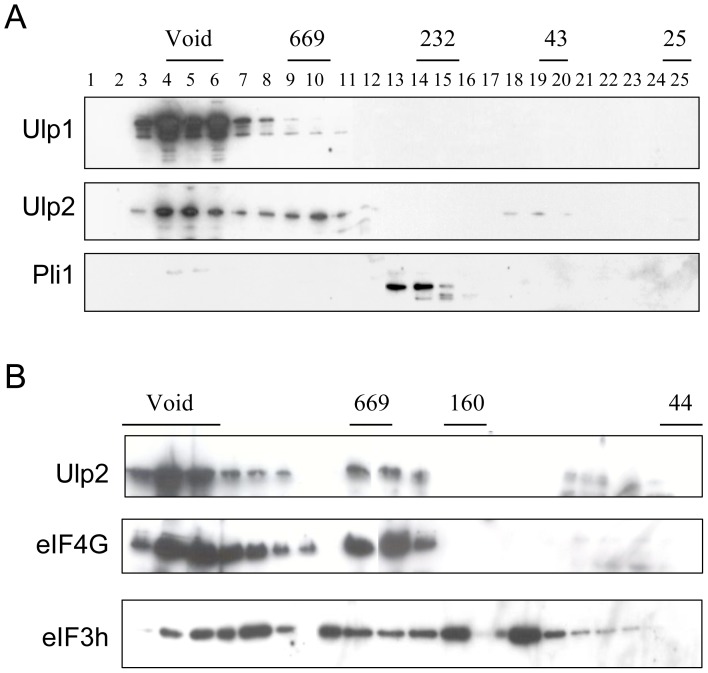
Ulp2 is present in high Mr complexes. Analysis of complexes by gel filtration. A. Total cell extracts from *ulp1-myc*, *ulp2-myc* or *pli1-myc* strains were analysed on a Sephadex 200 column, and fractions were western blotted with anti-myc antibodies. B. Total cell extracts from *ulp2-myc*,*eIF4G-HA* and *ulp2-myc*,*eIF3h-HA* strains were analysed on a Superose 6 column, fractions were western blotted with anti-myc and anti-HA antibodies.

Throughout most of the cell cycle, Ulp1 is associated with the nuclear envelope [13], and specifically with the nuclear pore complex [52], [53]. To determine whether Ulp2 is also part of a high Mr complex we undertook gel filtration analysis. Figure 2A indicates that, as expected, Ulp1 elutes in the void volume, consistent with it being present in a high Mr complex. Ulp2 also elutes in the void volume like Ulp1, but additionally, it is present in fractions corresponding to an approximate Mr of 670 kDa. This suggests that Ulp2 is likely to be present in at least two different complexes. In contrast to the results obtained for Ulp1 and Ulp2, Pli1, an E3 SUMO ligase [54], does not elute in these high Mr fractions, implying that it likely exists in cells as a monomer or possibly a dimer.

### Ulp2 is located in both the nucleus and cytoplasm, but is predominantly nuclear

**Figure 3:**
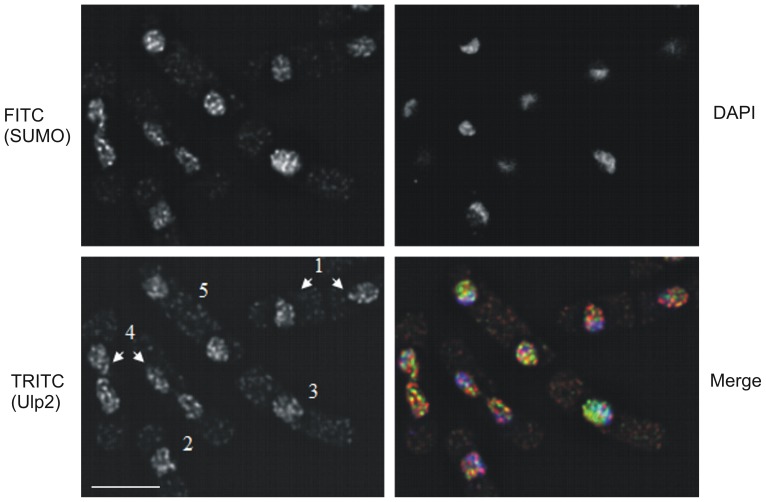
Ulp2 is localised predominantly within the nucleus. A. Cells containing myc-tagged *ulp2* as the sole copy of the *ulp2* gene were incubated with anti-myc antisera (mouse monoclonal) and anti-SUMO antisera (rabbit polyclonal) followed by TRITC-conjugated anti-mouse IgG antisera, FITC-conjugated anti-rabbit IgG antisera and DAPI. Merge  =  overlay of TRITC (red), FITC (green) and DAPI (blue) staining. 1: early G2 cells, 2,3: late G2 cells, 4: mitotic cells, 5: S phase cells. Bar  = 5 µm.

Since a proportion of Ulp2 co-elutes with Ulp1 in the void volume, we wished to determine whether some or all of the Ulp2 co-localises with Ulp1 in cells, i.e. is at the nuclear periphery. We therefore analysed the localisation of Ulp2. Figure 3 indicates that Ulp2 is present in foci that are predominantly nuclear, with a small proportion in the cytoplasm. Little if any Ulp2 is located at the nuclear periphery. Thus the location of Ulp2 is distinct from that of Ulp1 [13], indicating that it is unlikely to be part of nuclear pore complexes. In many cases, Ulp2 co-localises with SUMO. Ulp1 undergoes distinct changes in localisation during the cell cycle, its localisation changing from the nuclear periphery where it is for most of the cell cycle, to the region between the separating DNA masses during mitosis [13]. In contrast, the location of Ulp2 appears to be relatively unchanged in cells at different cell cycle stages. For example, during mitosis (Figure 3, TRITC panel, cells labelled 4), a time when Ulp1 relocalises, the distribution of intranuclear Ulp2 foci is very similar to that observed at other times in the cell cycle (cells labelled 1–3) and is unchanged.

### Ulp2 co-purifies with proteins associated with RNA metabolism and protein synthesis

**Figure 4:**
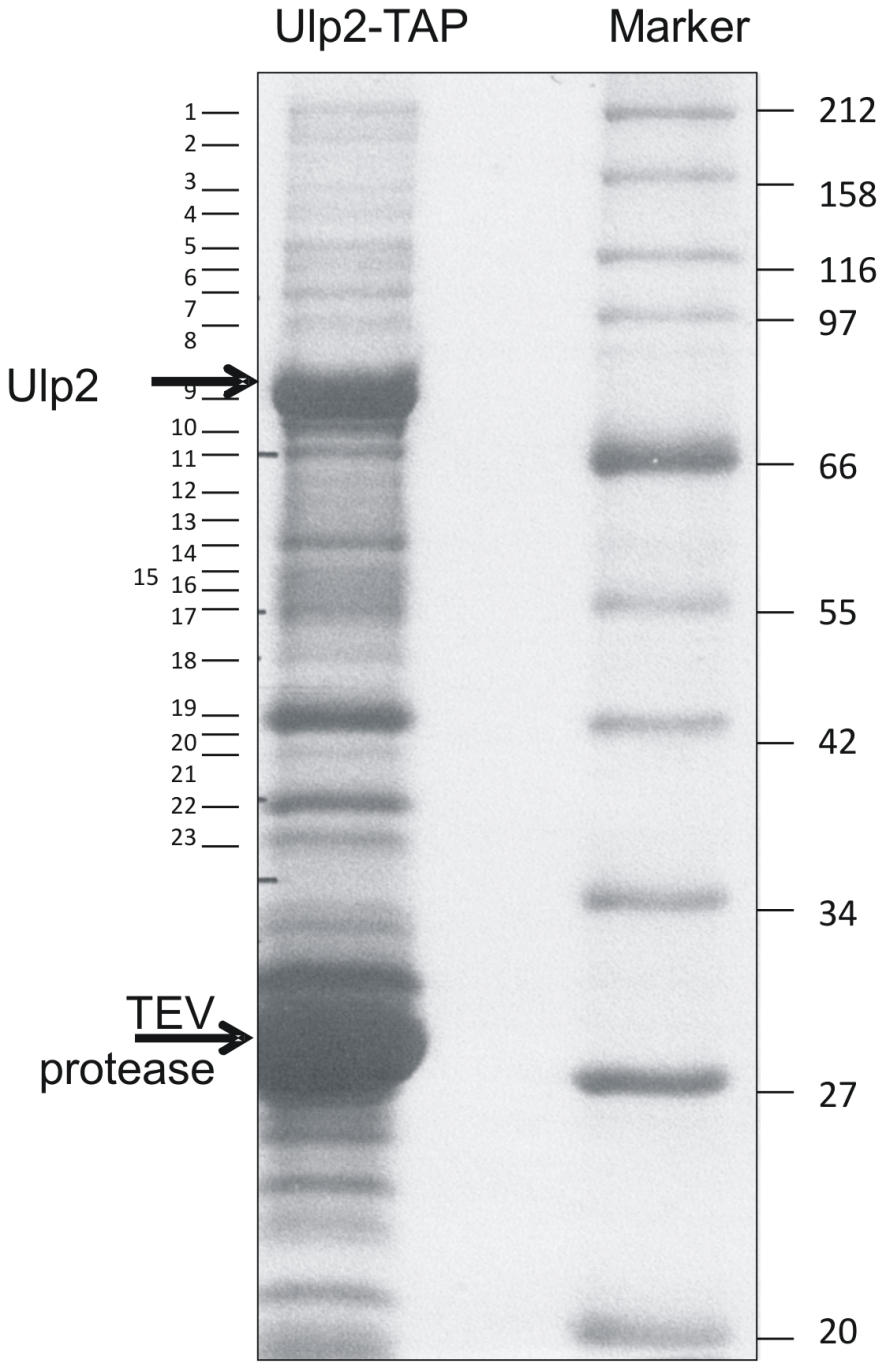
Purification of Ulp2-TAP. SDS-PAGE of Ulp2-Tap and associated proteins. TEV  =  TEV protease, used to cleave Ulp2 from TAP tag. Numbers refer to gel slices analysed by mass spectrometry.

To begin to identify the nature of the complexes observed in Figure 2, we C-terminally-tagged Ulp2 with TAP in the genome (*ulp2-TAP*) and isolated the tagged protein and associated proteins as described in Materials and Methods. Protein complexes were analysed by SDS PAGE (Figure 4) and fractions excised from the gel for mass spectrometric analysis. As shown in Table S1 and Table 2, the majority of the proteins identified are associated with RNA metabolism, such as RNA processing, ribosome biogenesis or initiation of translation. To ensure that these proteins co-purified specifically with Ulp2, a parallel purification was undertaken using Rad9-TAP, and from cells expressing the TAP tag alone (Figure S1). Rad9 is a member of the 9-1-1 complex required for the DNA integrity checkpoint [55], and would not be expected to interact with a the same proteins as those that interact with Ulp2. Very little protein co-purified with the TAP-tag alone, while purification of Rad9-TAP yielded a quite different set of bands. Most of the proteins co-purifying with Rad9 were associated with DNA metabolism as expected (data not shown) and only one protein, glyceraldehyde 3-phosphate dehydrogenase, was common to the Ulp2-TAP and Rad9-TAP preparations.

**Table 2:** Summary of proteins identified by mass spectrometry that co-purified with TAP-Ulp2.

Function	Protein
Translation	eIF2α, eIF2β, eIF3a, eIF3b, eIF3c, eIF3γ, eIF3h, eIF4G, EF1α EF2B, eEF3B, EF2, Pabp
RNA synthesis	Rpa1, Rpa2,
RNA processing	Rrp5, SPAC694.02, Exo2, Dhp1, Upf1, SPBC19G7.10C, Nop2, Dbp2, Prp19, Sla1,
Ribosome biogenesis	aconitate hydrolase/mitochondrial ribosomal protein subunit L49, SPAC22G7.05, SPAC1142.04(Noc2 predicted), Hsc1/Sks2, Rpl301, Rpl302, Rml2
DNA metabolism	Tcg1, Rfc5,
Other	Pfk1, SPBC16h5.12C, glutamate 5-kinase (predicted), Gpd1, Gpd3

Data from [6], [7], [23]–[26], [56]–[58].

A number of proteins required for ribosome biogenesis, including some of those we identified by mass spectrometry, have recently been demonstrated to be sumoylated (Table 2) [6], [7], [23]–[27], [56]–[58]. However, little is known about the effect of sumoylation on the function of translation factors. We therefore selected two translation initiation factors, eIF4G and eIF3h for further study. The analysis of some of the other factors will be described elsewhere. eIF4G has been well characterised in *S. cerevisiae* and mammalian cells [18], [22] and to some extent in *S. pombe* [59]. eIF4G acts as a scaffold protein as part of the eIF4F complex to recruit mRNA to the ribosome for translation [21], while eIF3h is a non-core subunit of the eIF3 complex linking eIF4F/mRNA to the ribosome in mammalian cells [60]. Gel filtration analysis of whole cell extracts from cells containing Ulp2-myc and either eIF4G-HA or eIF3h-HA indicates that the majority of eIF4G co-elutes with Ulp2 (Figure 2B). In contrast, eIF3h elutes in multiple fractions, suggesting it is present in several different sized complexes.

### eIF4G, but not eIF3h, is sumoylated in *S. pombe*

**Figure 5:**
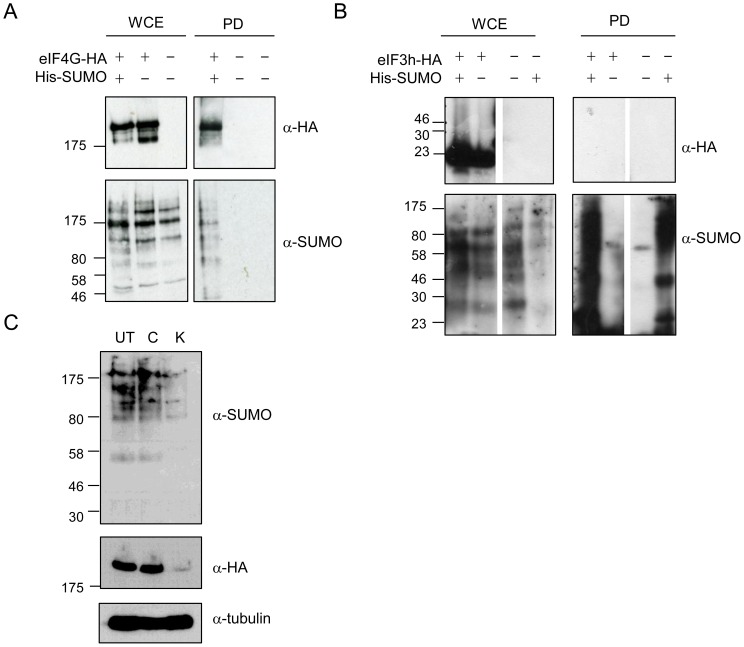
eIF4G, but not eIF3h, is sumoylated. His-tagged SUMO was expressed in cells containing genomically tagged (HA) copies of eIF4G (A) and eIF3h (B). WCE  =  whole cell extract, PD  =  Ni^2+^-agarose pull down. Blots were probed with anti-HA or anti-SUMO antisera. C. Western blot of whole cell extracts from cells containing genomically tagged eIF4G-HA. UT  =  untreated, C, K  =  incubated for 30 min with 100 µg/ml CHX (C) or 1 M KCl (K).

One possibility to explain the interaction of eIF4G and eIF3h with the SUMO protease Ulp2 is that they are themselves modified by SUMO. In order to determine whether this is the case, cells containing genomic copies of HA-tagged eIF4G or eIF3h were co-transformed with pREP41-His-SUMO. His-tagged SUMO was purified on Ni^2+^ agarose. Denaturing conditions (with 6 M guanidinium HCl in the binding buffer, followed by 6 M urea, 300 mM imidazole washes) were used to ensure that sumoylation of the individual proteins was being observed, rather than that of other components of the eIF4F or eIF3 complexes). Figure 5A, shows that eIF4G is specifically recovered in the presence of His-tagged SUMO (lane 1), but not in the absence of His-tagged SUMO (lane 2), indicating that it is sumoylated in *S. pombe*. In contrast, eIF3h is not recovered in either the absence or presence of His-tagged SUMO (Figure 5B), indicating that this translation factor is not sumoylated in fission yeast. Its co-purification with Ulp2 may thus be through the interaction of Ulp2 with other member(s) of the eIF3 complex.

### Conditions that induce stress granules affect the localisation and sumoylation of eIF4G

**Figure 6:**
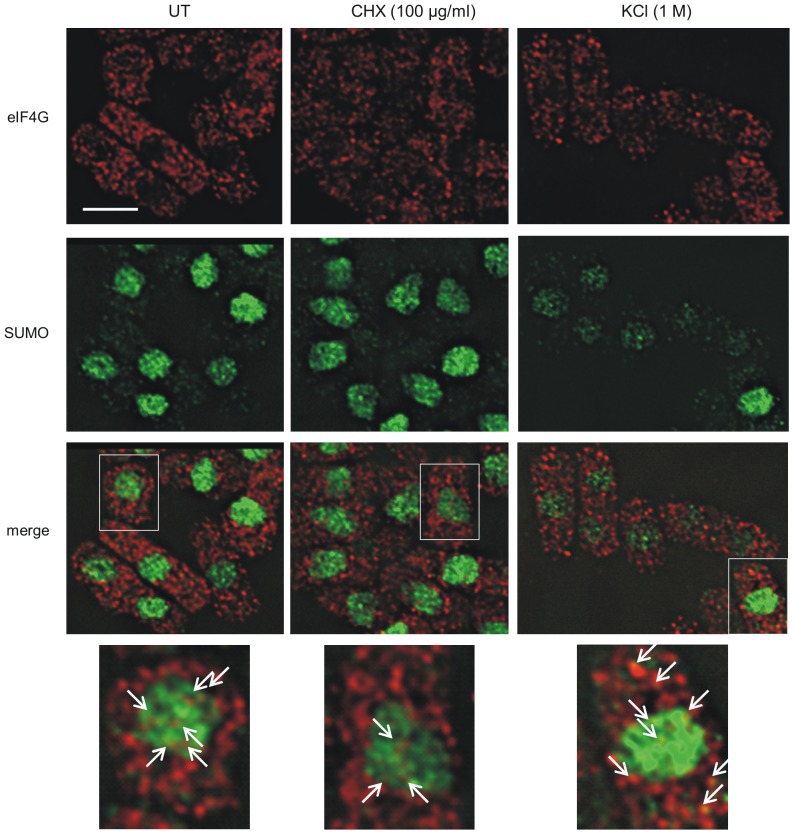
Effect of cycloheximide and KCl on localisation of eIF4G and SUMO. Cells containing eIF4G-HA, untreated (UT) or exposed to CHX (100 µg/ml) or KCl (1 M) as indicated, were incubated with anti-SUMO antisera (green) and anti-HA antisera (red). Bar  = 5 µm. Bottom panel, regions indicated by boxes in panel above. Arrows indicate sites of colocalisation of SUMO and eIF4G.

Since one of the functions of sumoylation is to affect protein localisation, we next investigated whether eIF4G and SUMO co-localise. Figure 6 shows that in untreated cells, as has been shown previously [61], the majority of eIF4G is cytoplasmic as expected for a translation initiation factor. As has been observed in *S. cerevisiae* and human cells [62], [63], a small amount of eIF4G is also present in the nucleus, where it is proposed to couple RNA processing events in the nucleus with translation in the cytoplasm. In contrast to the situation with eIF4G, the majority of the SUMO protein is present in the nucleus (Figures 3 and 6). We observe that a significant proportion of the nuclear eIF4G co-localises with SUMO, suggesting sumoylation of eIF4G may have a role in regulating RNA processing or localisation.

Protein synthesis can be inhibited by a variety of factors. For example, cycloheximide (CHX) interacts with ribosomes and inhibits the elongation step, while exposure of *S. pombe* cells to 1 M KCl inhibits protein synthesis by the sequestration of translation initiation factors and mRNA into cytoplasmic stress granules [64]. Following treatment with CHX, eIF4G staining is slightly more punctate than in untreated cells, while the pattern of SUMO staining is unchanged. In these cells, there is a low level of colocalisation of eIF4G and SUMO in the nucleus. Interestingly, exposure of cells to CHX results in distorted nuclei. The reason for this is not known, but it could be due to disruption of RNA processing and/or localisation by CHX.

In *S. pombe* and mammalian cells eIF4G and eIF4GI respectively, are among the translation factors present in stress granules [61], [65], [66]. To investigate stress granule formation in *S. pombe*, we exposed cells to 1 M KCl. In these cells, eIF4G is present in fewer, but quite bright, punctate cytoplasmic foci (Figure 6). This pattern of staining is similar to what has been observed for stress granules in *S. pombe*, and in particular, what has previously been observed for eIF4G in this organism [61], [64]. In these cells, there was occasional co-localisation of the two proteins in the cytoplasm and this appeared to reflect the appearance of eIF4G and SUMO in the same granule.

Another protein known to be present in stress granules is polyA-binding protein (PABP) [61]. We therefore compared the localisation of eIF4G and PABP in cells exposed to 1 M KCl. We observe PABP in large cytoplasmic granules, which are different to those we observe in cells only containing HA-tagged eIF4G-HA (Figure S2A and Figure 6). Curiously, in some of the cells that contain both eIF4G-HA and PABP-RFP, eIF4G is now also present in large granules where it co-localises with PABP. Further analysis of PABP-RFP containing cells indicated that a proportion of the SUMO is mislocalised to the cytoplasm (Figure S2B). This suggests that C-terminal RFP-tagging of PABP may affect its function and/or localisation.

Following exposure to 1 M KCl, we noticed that there was less staining of both eIF4G and SUMO compared to that in untreated cells. Western analysis of eIF4G and SUMO levels indicates that in response to 1 M KCl the levels of both proteins are significantly reduced (Figure 5C). The reason for this is unknown, but may be due to the fact that a proportion of the eIF4G and SUMO is insoluble and not recovered in the extract. Alternatively, and in our view the more likely explanation, we propose that in response to this stress, there is increased proteolysis of both proteins.

**Figure 7:**
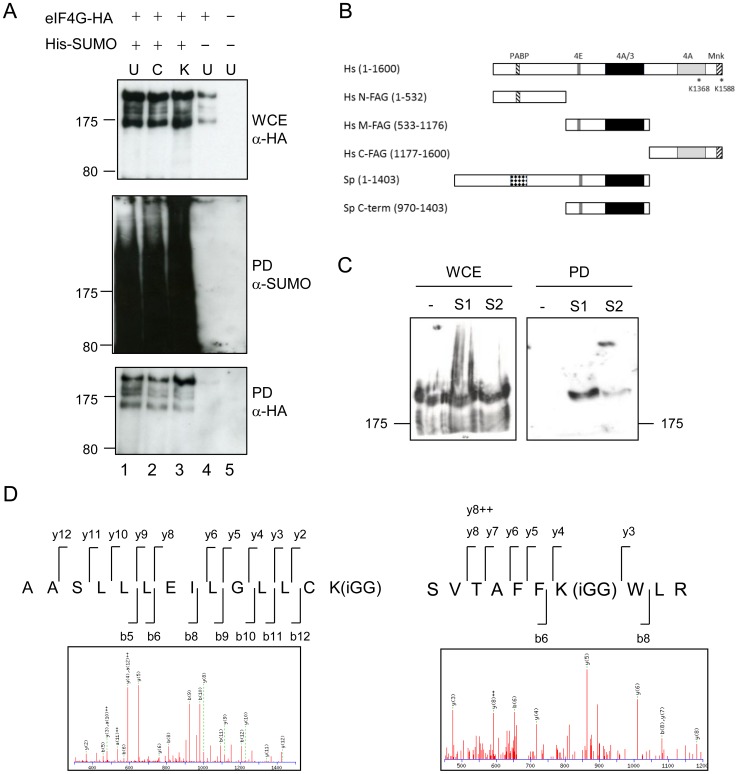
Human eIF4G is sumoylated. A. *S. pombe* cells containing His-tagged SUMO and HA-tagged eIF4G as indicated were treated with CHX (100 µg/ml) or KCl (1 M), and His-tagged SUMO pulled down, and analysed as in Figure 5. B. Comparison of human and eIF4G proteins, indicating protein binding domains: PABP =  polyA binding protein, 4E  =  eIF4E, 4A  =  eIF4A, 3 =  eIF3, Mnk  =  MAP kinase-interacting kinase 1. C. Whole cell extracts (WCE) and Ni^2+^ pull-down (PD) from extracts of HeLa cells stably transfected with His-tagged SUMO-1 (S1) or SUMO-2 (S2) or nothing (-). Western blots probed with anti-eIF4GI (KRERK epitope) antisera. D. Representative eIF4G ion mass spectra (MS/MS spectra) showing identification of the *in vitro* sites of sumoylation.

We next investigated whether sumoylation of eIF4G is affected by exposure of cells to either CHX (100 µg/ml) or KCl (1 M). Figure 7A indicates that there is an increase in sumoylation in response to KCl, with levels of sumoylation unaffected by exposure to CHX, when compared to levels in untreated cells (with relative levels being 1∶1∶1.5; wt, CHX-treated, KCl-treated, respectively). These data suggest that sumoylation of eIF4G may be associated with stress granule formation and/or proteolysis of the translation initiation factor.

### Human eIF4GI is sumoylated

In order to analyse the role of sumoylation of *S. pombe* eIF4G we investigated the possibility of testing the protein for ability to be sumoylated in our *in vitro* sumoylation assay, as this could help us identify the sumoylated lysine residue(s). However, two factors make this identification difficult. Firstly, in order to purify protein for an *in vitro* sumoylation assay, we would need to clone the full length *S. pombe* eIF4G cDNA. We have previously observed that plasmids containing the N-terminus of the *S. pombe* eIF4G coding sequence cannot be tolerated in *E. coli* [59], so that full length eIF4G cannot be expressed in *E. coli*. The reason for this is unknown, but may be due to the presence of a highly repeated sequence within the eIF4G coding sequence. Secondly, this highly repeated sequence (present in the coding sequence in the *S. pombe*, but not in the *S. cerevisiae* or human proteins) contains 16 repeats of a perfect sumoylation site consensus motif (AKRE), which would likely make identification of the site(s) difficult, even if we were able to express the full length protein. We therefore expressed a C-terminal fragment (comprising aa 970–1403), which contains eIF4E, eIF4A and eIF3 binding sites (Figure 7B) and tested this in our *in vitro* sumoylation assay. We did not observe any sumoylation of this fragment, implying that sumoylation likely occurs in the N-terminus of the protein.

To further analyse the role of sumoylation we set out to determine whether human eIF4GI is sumoylated and if so, to identify the sumoylation site(s) in this protein. We used HeLa cell lines stably transfected with either His-SUMO-1 or His-SUMO-2 [38], [39]. His-tagged SUMO was recovered from cell extracts prepared under denaturing conditions. Figure 7C indicates that eIF4GI is not recovered from extracts of cells that do not contain His-tagged SUMO (lane1), but is isolated from extracts of cells containing His-SUMO-1 (lane 2) and to a lesser extent from cells expressing His-SUMO-2 (lane 3). This confirms that, like *S. pombe* eIF4G, human eIF4GI is sumoylated.

We next sought to identify the sumoylation sites on human eIF4GI. In order to facilitate our analysis, we used three different human eIF4G fragments, N-FAG, M-FAG and C-FAG (Figure 7B, [37]). These protein fragments were purified from *E. coli* and tested in our *in vitro* sumoylation assay (data not shown). Slow migrating forms of eIF4G were excised from gels and analysed by mass spectrometry. Two sumoylation sites were identified: K1368 and K1588 (Figure 7D). These map to two domains of eIF4GI which interact with eIF4A and the protein kinase, Mnk1, respectively [18], [21], [22]. These results suggest that sumoylation may affect the interaction of eIF4GI with these two proteins.

## Discussion

In order to analyse the role of *S. pombe* Ulp2, we purified Ulp2-TAP-containing complexes. We identified proteins involved in RNA synthesis or processing, ribosome biogenesis and translation. This is consistent with recent reports that a number of proteins required for ribosome biogenesis and RNA processing are sumoylated [6], [67], [68]. While this manuscript was in preparation, a global analysis of the SUMO system interactome in *S. cerevisiae* identified a range of proteins including a number required for ribosome biogenesis and rRNA processing that interact with Ulp2 [69]. Additionally, the nucleolar SUMO-specific protease, SENP3, has been demonstrated to reverse the SUMO modification of nucleophosmin to be required for rRNA processing [70].

Although a number of translation factors, required for both the initiation and elongation steps of protein synthesis, have been identified in global screens as being sumoylated e.g. [23]–[28], [30], [31], little is known about the role of sumoylation of these proteins. This is in contrast to the situation with the role of sumoylation in ribosome biogenesis. We therefore focussed our attention on two *S. pombe* translation initiation factors in our list of Ulp2-interactors: eIF4G and eIF3h. Both proteins are known to be present in high Mr complexes, with eIF4G being part of the eIF4F complex while eIF3h is part of the eIF3 complex [22]. We demonstrate here that eIF4G, but not eIF3h is sumoylated *in vivo*. These results are supported by the genome-wide analyses of sumoylated proteins that have been undertaken, that indicate that eIF4G is sumoylated [24], [28] but which have not to date identified eIF3h as a sumoylation target.

As the most prominent role of translation initiation factors is in cytoplasmic protein synthesis, we began by investigating whether Ulp2 is associated with polysomes. However, we observed that while Ulp2 migrated at the same position in sucrose gradients as polysomes, it was still present in these fractions under conditions (2.5 mM EDTA) where polysomes were disrupted, indicating that the majority of Ulp2 is not associated with actively translating polysomes (data not shown). This result confirms our gel filtration analysis and localisation studies, and indicates that Ulp2 is present in very high molecular weight complexes, but discounts the possibility that Ulp2 is associated with actively translating polysomes.

The role of sumoylation of translation factors has not been well studied, apart from that of eIF4E [29], [33]. eIF4E is an mRNA cap-binding protein, and one of the proteins that interacts with eIF4G to form the eIF4F complex [22]. eIF4E is regulated by phosphorylation and by interaction with eIF4E-binding proteins (4E-BPs). Sumoylation of eIF4E on five lysines is promoted by its phosphorylation at S209, and results in its dissociation from 4E-BP1. Sumoylation did not interfere with mRNA recognition but enhanced eIF4F complex assembly on the mRNA cap, promoting the expression of ornithine decarboxylase, c-myc and Bcl-2, driving the anti-apoptotic and oncogenic activity of eIF4E [33]. As phosphorylation of eIF4E has been shown to play a role in selective nuclear export of mRNA [71], it is likely that sumoylation of eIF4E occurs in the nucleus and/or as it emerges into the cytoplasm

We have shown that in response to osmotic stress (1 M KCl), conditions that induce stress granules in fission yeast, the overall levels of SUMO and eIF4G are reduced. We have also shown that under these conditions, there is increased sumoylation of eIF4G. The role of this modification is not known. Our results suggest two possible scenarios: the first being that sumoylation is targeting eIF4G for degradation, possibly via the action of a SUMO-targeted ubiquitin ligase (STUbL). The second possibility is that sumoylation may be targeting eIF4G to stress granules. Further work is needed to distinguish between these two possibilities.

The two sumoylation sites in human eIF4GI that we have identified are not conserved in fission yeast eIF4G, as this protein lacks the C-terminal domains present in human eIF4GI (Figure 7B). Their positions suggest that sumoylation of this protein may be affecting interactions of eIF4GI with eIF4A and Mnk1. eIF4A is a DEAD-box protein that participates in translation initiation and binds to eIF4GI [18], [21], [22]. Functioning as an ATP-dependent RNA helicase, eIF4A is believed to unwind secondary structure in the 5′-untranslated region of mRNAs to enable ribosome scanning. The RNA-stimulated ATPase and ATP-dependent helicase activities of eIF4A are enhanced by its interaction with two domains on eIF4GI, one in the C-terminus and one in the middle domain [18], [22]. Interaction and subsequent recycling of eIF4A from the eIF4G/eIF4A complex stimulates the eIF4A helicase activity required for the mRNA scanning process. It is possible that sumoylation of eIF4GI either directly or indirectly affects the interaction with eIF4A, thereby regulating translation initiation. Mnk1 is a kinase which binds at the extreme C-terminus of eIF4GI and regulates the phosphorylation of eIF4E at Ser209 [18], [21], [22]. Phosphorylated eIF4E has been shown to be modified by sumoylation on five lysine residues [33] promoting eIF4F complex formation and specific protein synthesis [33]. Sumoylation of K1588 on eIF4GI could prevent the binding of Mnk1, reduce eIF4E phoshorylation and thereby abrogate sumoylation of eIF4E and specific mRNA translation. As phosphorylation of eIF4E is associated with tumour cell formation and increased resistance of tumour cells to apoptosis, sumoylation of eIF4GI at this site could provide a novel and undiscovered mechanism to regulate cell growth and proliferation in mammalian cells. Further work needs to be done to address this.

In conclusion, our results demonstrate that *S. pombe* and human eIF4GI are both sumoylated, and that in *S. pombe* this modification is increased under conditions that promote the formation of stress granules. We have also identified the target lysine residues that are used for sumoylation *in vitro* in human eIF4GI. It will be of interest to determine whether these sites are also used *in vivo*, and to identify the role of this sumoylation.

## Supporting Information

Figure S1 **Comparison of proteins co-purifying with Ulp2-Tap and Rad9-Tap.** Extracts from cells expressing Ulp2-Tap, Rad9-Tap (Methods S1) or Tap alone were subjected to the same purification procedure and analysed by SDS-PAGE followed by staining with colloidal coommassie.
(TIF)

Figure S2 **Colocalisation of eIF4G with PABP.** A. Strain containing eIF4G-HA and PABP-RFP stained with anti-HA and anti-RFP antisera. Secondary antisera: anti-rabbit FITC conjugated, anti-mouse TRITC-conjugated. B. Strains containing either eIF4G-HA or Pabp-RFP (Methods S1) as indicated, stained with anti-SUMO antisera.
(TIF)

Table S1 **Identity of proteins co-purifying with Ulp2-TAP.** Proteins identified by LC MS/MS (Methods S1).
(DOCX)

Methods S1 (DOCX)
