# Supplementary figures and images for: The S. pombe Translation Initiation Factor eIF4G Is Sumoylated and Associates with the SUMO Protease Ulp2
Source: PLoS One. 2014 May 12;9(5):e94182. doi: 10.1371/journal.pone.0094182 (PMC4018355; doi:10.1371/journal.pone.0094182)

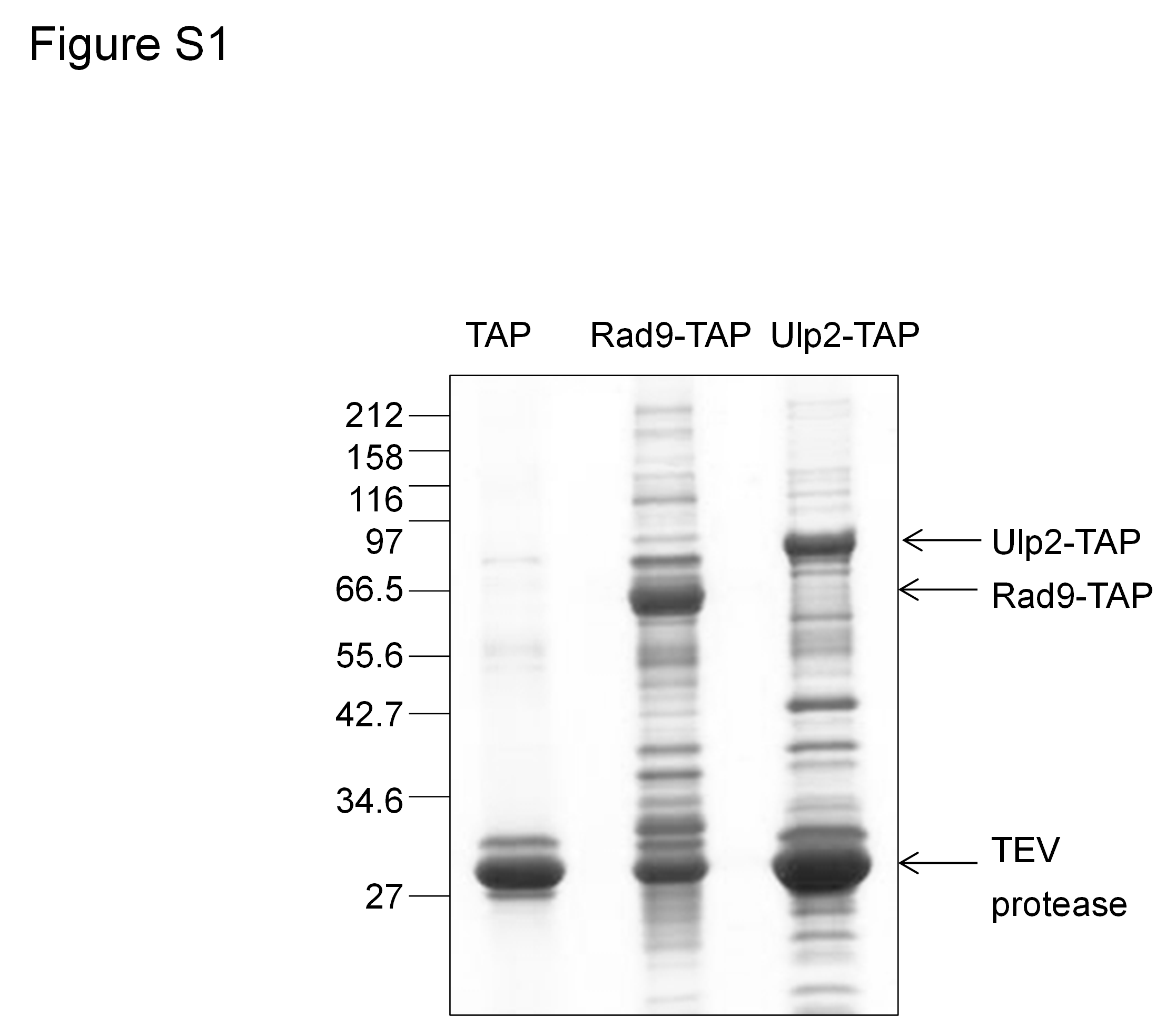

Supplement: Figure S1 — Comparison of proteins co-purifying with Ulp2-Tap and Rad9-Tap. Extracts from cells expressing Ulp2-Tap, Rad9-Tap (Methods S1) or Tap alone were subjected to the same purification procedure and analysed by SDS-PAGE followed by staining with colloidal coommassie. (TIF) [file pone.0094182.s001.tif]

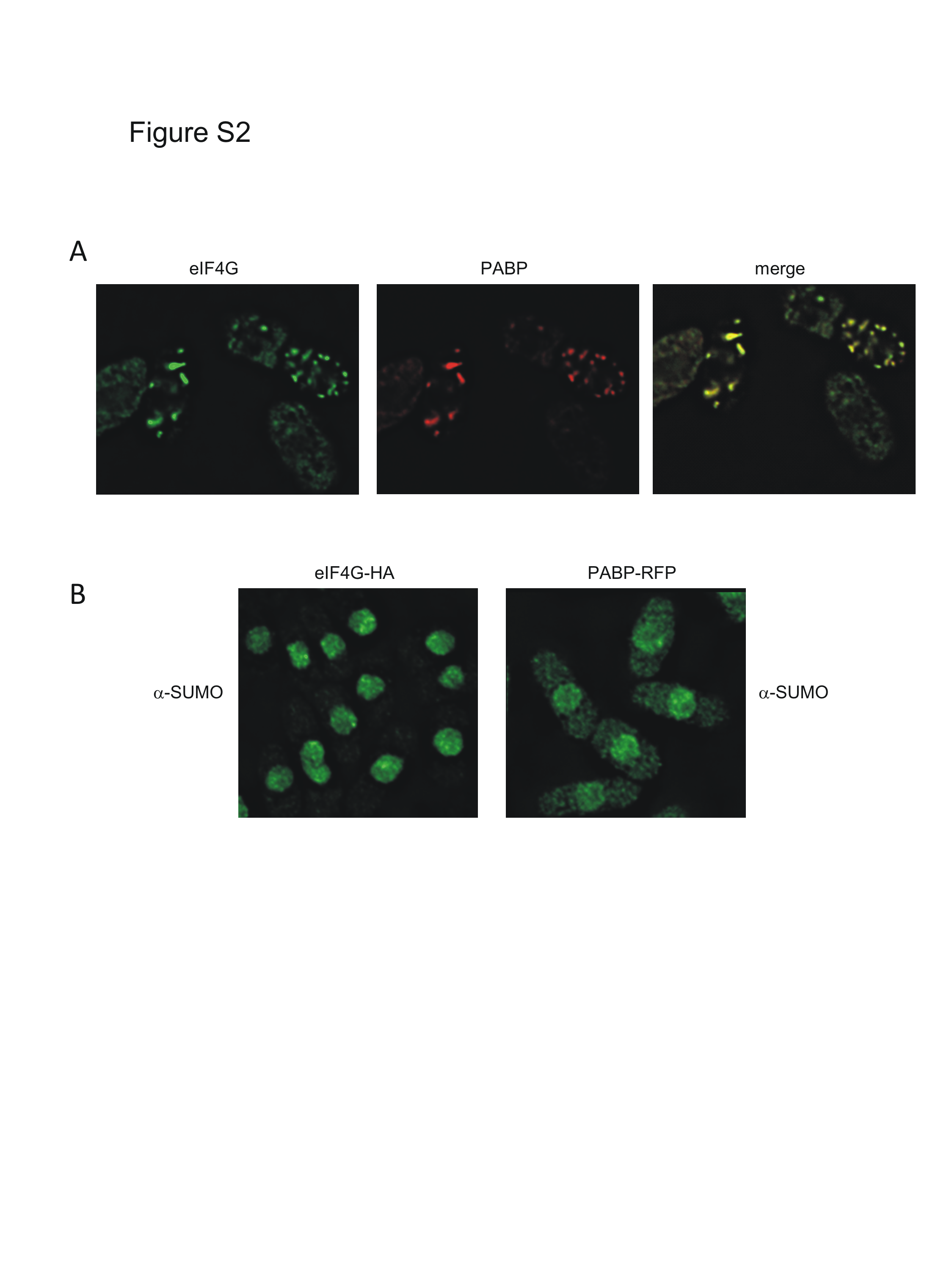

Supplement: Figure S2 — Colocalisation of eIF4G with PABP. A. Strain containing eIF4G-HA and PABP-RFP stained with anti-HA and anti-RFP antisera. Secondary antisera: anti-rabbit FITC conjugated, anti-mouse TRITC-conjugated. B. Strains containing either eIF4G-HA or Pabp-RFP (Methods S1) as indicated, stained with anti-SUMO antisera. (TIF) [file pone.0094182.s002.tif]
