# Supplementary material for: The S. pombe Translation Initiation Factor eIF4G Is Sumoylated and Associates with the SUMO Protease Ulp2
Source: PLoS One. 2014 May 12;9(5):e94182. doi: 10.1371/journal.pone.0094182 (PMC4018355; doi:10.1371/journal.pone.0094182)
Supplement: Table S1 — Identity of proteins co-purifying with Ulp2-TAP. Proteins identified by LC MS/MS (Methods S1). (DOCX) [file pone.0094182.s003.docx]

**Table S1**

**Identity of proteins co-purifying with Ulp2-TAP**

| **Gel slice** | **Systematic name** | **Gene name** |  |
| --- | --- | --- | --- |
| 1 | SPAC17C9.03 | tif471 | eIF4G, translation initiation factor |
| 2 | SPBC4C3.05 | nuc1 | Rpa1, DNA-directed RNA polymerase I, large subunit |
| 2 | SPC1183.07 | rrp5 | U3 sno-RNP-associated protein (Rrp5) (predicted) |
| 2 | SPAC694.02 |  | DEAD/DEAH box helicase |
| 3 | SPAC17A5.14 | exo2 | Exonuclease II (Pacman) |
| 4 | SPBP23A10.07 | rpa2 | DNA-directed RNA polymerase subunit, Rpa2 |
| 4 | SPAC26A3.12c | dhp1 | 5’-3’ exonuclease |
| 5 | SPCC417.08 | tef3 | Translation elongation factor eEF3 |
| 6 | SPBC17D11.05 | tif32 | Translation initiation factor eIF3a |
| 6 | SPAC4A8.16c | tif33 | Translation initiation factor eIF3c |
| 7 | SPBC16H5.02 | pfk1 | 6-phosphofuctokinase (predicted) |
| 7 | SPAC16C9.06 | upf1 | ATP-dependent RNA helicase |
| 7 | SPAC513.01c | eft201 | Translation elongation factor2 (EF-2) |
| 7 | SPCP31B10.07 | eft202 | Translation elongation factor (EF-2) |
| 7 | SPBP4H10.15 |  | Aconitate hydratase/mt ribosomal protein subunit L49 (predicted) |
| 7 | SPAC17A5.07c | ulp2 | SUMO protease |
| 8 | SPAC25G10.08 |  | Translation initiation factor eIF3b |
| 8 | SPBC19G7.10C |  | Topoisomerase II-associated deadenylation-dependent mRNA decapping factor |
| 8 | SPBP8B7.20c |  | RNA methyl transferase Nop2 (predicted) |
| 8 | SPAC22G7.05 |  | krr family protein (predicted) |
| 8 | SPAC17A5.07c | ulp2 | SUMO protease |
| 8 | SPAC1142.04 | noc2 | Noc complex subunit family (predicted) |
| 8 | SPBC16H5.12C |  | KRR family protein (predicted) |
| 8 | SPAC57A7.04 | pabp | Poly-A binding protein |
| 11 | SPBC1709.05 | sks2/hsc1 | Ribosome-associated molecular chaperone |
| 11 | SPAC17A5.07c | ulp2 | SUMO protease |
| 14 | SPBC800.08 | gcd10 | eIF3-γ (predicted) |
| 14 | SPBP8B7.16C | dbp2 | ATP-dependent RNA helicase |
| 14 | SPAC17A5.07c | ulp2 | SUMO protease |
| 17 | SPBC839.15C | ef1a-c | Translation elongation factor EF-1α |
| 17 | SPCC794.09C | ef1a-a | Translation elongation factor EF-1α |
| 17 | SPAC23A1.10 | ef1a-b | Translation elongation factor EF-1α |
| 17 | SPBC660.11 | tcg1 | Single-stranded telomeric binding protein |
| 17 | SPAC29A4.08C | prp19 | Ubiquitin protein ligase E4 |
| 19 | SPAC57A10.10C | sla1 | La protein homolog |
| 19 | SPAC17A5.07c | ulp2 | SUMO protease |
| 20 | SPAC17A5.03 | rpl301 | 60S ribosomal protein L3 |
| 20 | SPAPB8E5.06C | rpl302 | 60S ribosomal protein L3 |
| 20 | SPAC821.05 |  | eIF3h |
| 20 | SPAC32A11.04C | tif212 | Translation initiation factor eIF2β (predicted) |
| 20 | SPAC17H9.13C |  | Glutamate 5-kinase (predicted) |
| 20 | SPAC57A10.10C | sla1 | La protein homologue |
| 20 | SPAC17A5.07c | ulp2 | SUMO protease |
| 22 | SPBC83.14C | rfc5 | DNA replication factor C complex subunit |
| 22 | SPBC32F12.11 | tdh1 | Glyceraldeyde-3-phosphate dehydrogenase |
| 22 | SPAC17A5.07c | ulp2 | SUMO protease |
| 22 | SPCC16C4.15 | rml2 | Mitochondrial ribosomal protein subunit L2 (predicted) |
| 23 | SPBC354.12 | gpd3 | Glyceraldehyde 3-phosphate dehydrogenase |
| 22 | SPAC3G9.09C | tif211 | Translation initiation factor eIF2α |
| 23 | SPAC17A5.07c | ulp2 | SUMO protease |
