# Supplementary material for: The S. pombe Translation Initiation Factor eIF4G Is Sumoylated and Associates with the SUMO Protease Ulp2
Source: PLoS One. 2014 May 12;9(5):e94182. doi: 10.1371/journal.pone.0094182 (PMC4018355; doi:10.1371/journal.pone.0094182)
Supplement: Methods S1 — (DOCX) [file pone.0094182.s004.docx]

**Methods S1**

**Strains**

*rad9-TAP*, *ade6-704*, *leu1-32*, *ura4-D18*, *h^-^* was from A.M. Carr (University of Sussex), *pabp-RFP:kan*, *h*^-^ was from P. Sunnerhagen (University of Gothenburg) [61].

**Antibodies**

Anti-RFP monoclonal antibody was from Abcam (Ab 62341), and rabbit anti-HA antisera were from Sigma (H6908).

**Mass spectrometry**

Bands excised from gel separating TAP-Rad9 associated proteins and TAP-Ulp2 (from the same preparation as used in main text) were analysed by mass spectrometry (D Stead, University of Aberdeen, L.D.B. University of Sussex).
